# Two Cases of Cutaneous Mycobacterium szulgai Infection

**DOI:** 10.7759/cureus.25070

**Published:** 2022-05-17

**Authors:** Larimar Rodriguez, Kory Lee, Charles Phillips

**Affiliations:** 1 Dermatology, University of New Mexico School of Medicine, Albuquerque, USA; 2 Dermatology, Raymond G. Murphy Department of Veterans Affairs Medical Center, Albuquerque, USA

**Keywords:** nonhealing rash, skin rash, mycobacteria, infectious disease, nontuberculous mycobacteria, ntm, mycobacterium szulgai

## Abstract

Although nontuberculous mycobacteria (NTM) infection is often thought of as a pulmonary disease, it can manifest on the skin in rare cases. In this report, we describe two cases of cutaneous *Mycobacterium szulgai* that presented as nonhealing rashes that were initially thought to be caused by bacterial cellulitis but did not respond to broad-spectrum antibiotics. In both cases, the rash began on the upper extremities. We believe that these cases will be of interest as they demonstrate a rare cutaneous infection and an unusual presentation for NTM. It is important to consider NTM, and *M. szulgai *specifically, in an immunocompromised patient with a nonhealing rash and initiate appropriate diagnostic studies.

## Introduction

*Mycobacterium szulgai* is a slow-growing scotochromogenic nontuberculous mycobacterium (NTM) that is very rare in humans [1,2]. *M. szulgai* most commonly presents as pulmonary disease, similar to *Mycobacterium tuberculosis*, but can have extrapulmonary manifestations as well. While cutaneous reports of *M. szulgai* are limited in the literature, one commonality of existing cases is the association with immunosuppression secondary to medication or active disease [1,3-5]. In this report, we describe two patients with nonhealing rashes initially thought to be cellulitis and unresponsive to broad-spectrum antibiotics. Specific treatment with a multi-drug regimen was started in both patients and, within one to two months, both had significant improvement. It is important for clinicians to consider NTM, including *M. szulgai*, in the setting of nonhealing wounds and soft tissue infections as diagnosis requires molecular detection, and proper treatment should not be delayed. 

## Case presentation

Case 1 

**Figure 1:**
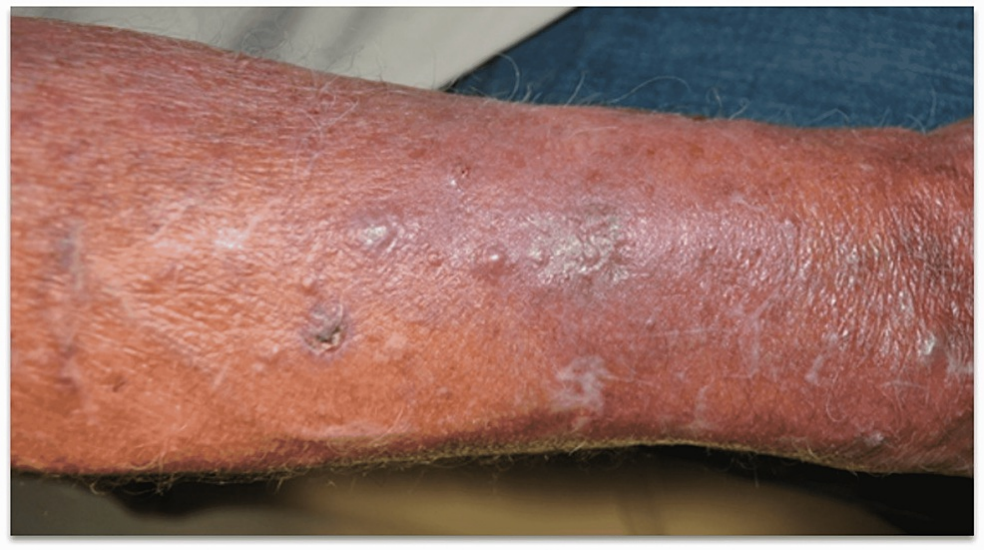
Clinical presentation, Case 1: Erythematous, edematous plaques with intermixed papules and nodules, with surrounding erythema over the right forearm. Focal necrosis and crusting seen are due to biopsy site change.

A 72-year-old male presented with a three-month history of a rash that started on the right proximal forearm. When the rash first arose, he had an associated fever that had been resolved by the time of presentation. A physical exam revealed erythematous, edematous plaques with intermixed papules, nodules, and milia cysts with surrounding erythema over the right forearm (Figure 1). He had a history of chronic rheumatoid arthritis and had been taking adalimumab and methotrexate for three years. The rash was initially treated as cellulitis with no response to cephalexin and trimethoprim-sulfamethoxazole.

**Figure 2:**
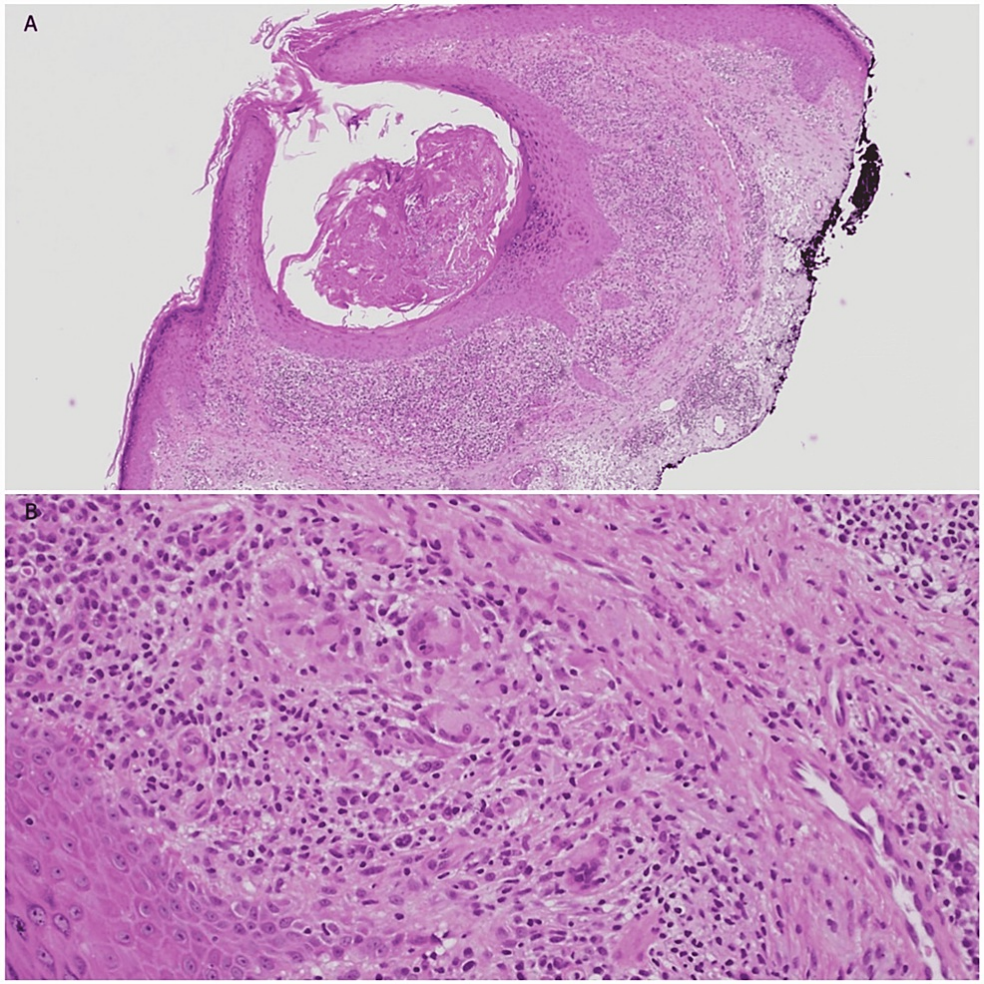
Microscopic examination of skin biopsy in Case 1: (A) Focally invaginated epidermis with acanthosis; (B) Granulomatous inflammation without necrosis and giant cells.

A punch biopsy was performed and histologic evaluation found a focally invaginated epidermis with acanthosis, granulomatous inflammation without necrosis, giant cells, plasma cells, and a single acid-fast organism (Figure 2). The tissue was sent to the University of Washington, Seattle, Washington, United States, for polymerase chain reaction (PCR) testing for mycobacteria, and *M. szulgai* was identified by high-performance liquid chromatography. 

**Figure 3:**
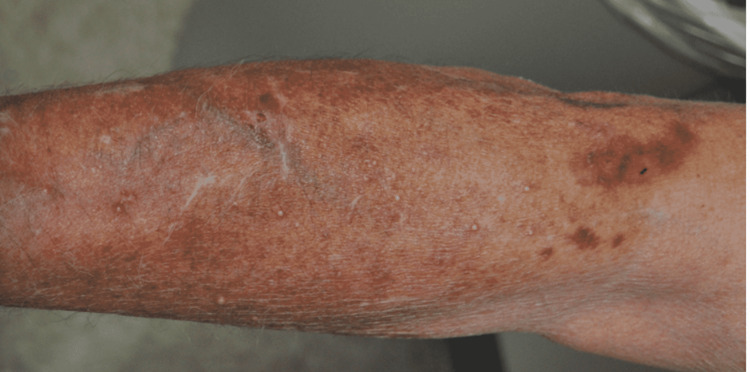
Clinical presentation, Case 1: There was clinical improvement after one month of treatment with less erythema and increased milia in previously affected areas.

The patient continued methotrexate, discontinued adalimumab, and in just under three weeks there was a slight improvement in his rash without targeted antimicrobial therapies. He was then started on a 12-month treatment regimen of azithromycin (500mg/d), rifampin (600mg/d), and ethambutol (15mg/kg/d). A chest x-ray was performed and showed no concern for disseminated infection. Follow-up after one month of treatment demonstrated a significant reduction of the erythema, papules, and nodules with an increase in milia over the affected areas (Figure 3). A direct cause of the patient's infection remains unclear. 

Case 2

**Figure 4:**
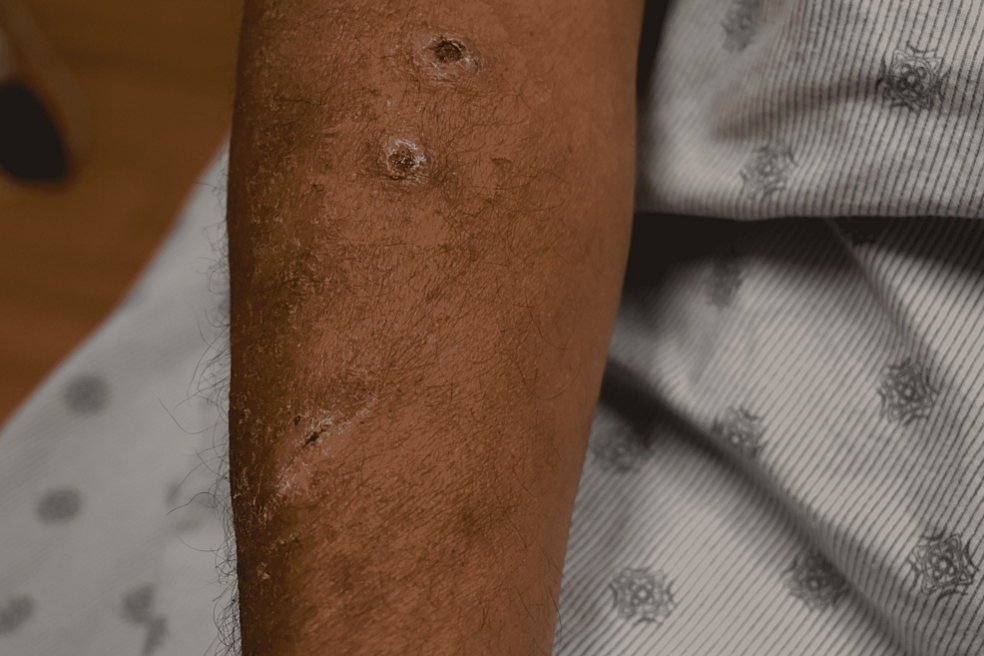
Clinical presentation, Case 2: Eschar-ulcerative lesions of the right forearm with surrounding erythema in a roughly linear lymph nodal distribution.

**Figure 5:**
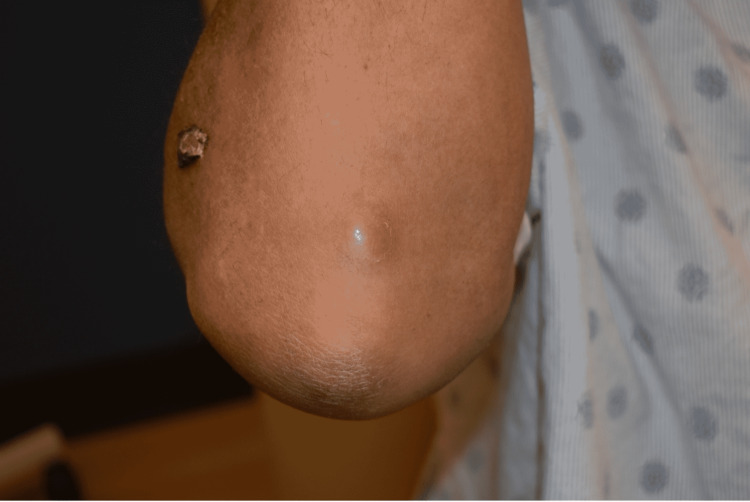
Clinical presentation, Case 2: A nodular lesion with erythema was found over the right elbow and another was present over the left forearm (not shown).

A 57-year-old male presented with a two-month history of a rash over his right forearm and a two-week history of dark spots he had noticed in his mouth. The rash began on his right index finger and progressed to his arm. A physical exam revealed eschar-ulcerative lesions of the right forearm with surrounding erythema in a roughly linear lymphatic distribution (Figure 4). A nodular lesion with erythema was found over the right elbow and another over the left extensor forearm (Figure 5). Discrete, hyperpigmented, violaceous lesions of the tongue and hard palate were also observed. The patient had a history of diabetes, chronic kidney disease, liver transplant six years prior, and recurrent cryptococcal meningitis on lifelong fluconazole. He had also been taking prednisone and tacrolimus following his transplant. He was seen two months prior by urgent care for the finger rash and was treated with cephalexin and trimethoprim-sulfamethoxazole but had no resolution. He works in a field and trims roses seasonally, and has several animals at home, including a cat, dogs, cows, and several small fish. The patient had labs done on admission, which showed neutropenia.

A punch biopsy of the upper extremity was performed and demonstrated acid-fast bacilli and gram-positive beaded filamentous rods within suppurative granulomatous inflammation. The tissue culture grew *M. szulgai*. There was concern for *Nocardia *infection, and a brain MRI was performed to rule out disseminated disease, which was unremarkable. An MRI of the right arm demonstrated a small volume abscess and showed no evidence of fascial inflammation. A tongue biopsy revealed hyperplastic and parakeratotic squamous mucosa exhibiting pigmented melanophages within the intraepithelial capillaries. The tongue biopsy was inconclusive but suggested a prior trauma to the area as opposed to infection by M. szulgai. It is likely that the patient acquired the M. szulgai infection from cleaning his fish tank.

He was started on empiric antibiotics until the final acid-fast culture results were received, when he was switched to azithromycin (500mg/d), isoniazid/pyridoxine (300mg/d;50mg/d), and ethambutol (1600mg/d). His tacrolimus dose was changed to once a day instead of twice a day as recommended by his transplant team. Weekly complete blood count (CBC), comprehensive metabolic panel (CMP), tacrolimus trough, EKGs for QT corrected for heart rate (QTc), and ethambutol levels were recommended due to the patient's extensive medication list. Follow-up after seven weeks of treatment showed significant improvement of the skin lesions, with post-inflammatory hyperpigmentation and no erythema or nodules. At the nine-week follow-up appointment, the patient complained of right hip pain, and an MRI was performed, showing a patchy area of abnormal bone marrow that could represent marrow stimulation. The patient continues to be on the multi-drug regimen with no end-of-treatment date planned due to the possibility of disseminated *M. szulgai *infection affecting the bone marrow and is pending further work-up.

## Discussion

While there has been an increase in the reported cases of *M. szulgai* infection, it is still considered a rare human pathogen [1,2]. *M. szulgai* has been recovered from environmental sources including snails, aquarium water, swimming pool water, and tropical fish [6]. There is no evidence of human-to-human transmission. The first finding of the pathogen reported in the literature was in 1972, and fewer than 15 total cases of cutaneous infection due to *M. Szulgai* have been published in the literature in English [7].

Clinicians face many challenges in the prompt diagnosis of cutaneous *M. szulgai* as skin lesions are nonspecific and can be confused for cellulitis. Clinical findings reported of *M. szulgai* include boggy, tender, erythematous nodules, which are indurated, minimally tender, and occasionally ulcerated. Histologic findings include chronic inflammation with mixed cellular infiltration, epithelioid granulomas without caseation, and zero to many acid-fast bacilli (AFB) [1].

While most of the reported cases of *M. szulgai* include patients who are immunocompromised, a small number of the reported infections occurred in seemingly fully immunocompetent people [8-10]. Both of the patients we presented were immunocompromised and undergoing immune-modulating therapy. Thus, clinicians should include *M. szulgai* in the differential diagnosis in the setting of lesions nonresponsive to antibiotics in immunosuppressed patients.

Once NTM infection is suspected clinically, it is important to obtain biopsies and tissue cultures to establish a diagnosis. Although there are no clear guidelines for the treatment of *M. szulgai*, it typically has susceptibility patterns paralleling those of *M. tuberculosis* and is treated similarly with a multiple-drug regimen, though with the addition of a macrolide. Our patients were treated with azithromycin, ethambutol, and either rifampin or isoniazid/pyridoxine.

## Conclusions

These cases highlight the importance of considering NTM infections with a nonhealing rash in immunocompromised patients. The presentation is non-specific and is often confused with cellulitis. We hope that these two cases increase awareness of the clinical presentations of *M. szulgai*, as early clinical suspicion will expedite appropriate diagnostic studies and treatment, avoiding redundant antibiotic treatment.
